# ⁶⁸Ga-FAPI PET/CT reveals fibroblast activation beyond biochemical fibrosis scores in chronic liver disease

**DOI:** 10.1186/s41824-026-00336-1

**Published:** 2026-11-13

**Authors:** Surya Pratap Singh, Amit Goel, Prabhakar Mishra, Manish Dixit, Aftab Hasan Nazar, Manish Ora, Prasanta Kumar Pradhan, Sanjay Gambhir

**Affiliations:** 1 Department of Nuclear Medicine SGPGIMS, Lucknow, 226014 India; 2 Department of Hepatology SGPGIMS, Lucknow, 226014 India; 3 Department of Biostatistics & Health Informatics SGPGIMS, Lucknow, 226014 India; 4 Department of Nuclear Medicine, Hind Institute of Medical Science, Barabanki, Uttar Pradesh 225003 India

**Keywords:** Fibroblast activation protein, FAPI PET, Chronic liver disease, Liver fibrosis, Molecular imaging, Fibrogenesis

## Abstract

**Purpose:**

To evaluate the role of ⁶⁸Ga-FAPI PET/CT in assessing fibroblast activity in patients with chronic liver disease (CLD). We studied FAPI liver uptake and its relationship with functional disease severity (Child-Pugh score) and biochemical fibrosis indices (APRI and FIB-4).

**Methods:**

This prospective study included 10 control subjects without liver disease and 33 patients with CLD across Child–Pugh classes A–C. Biochemical evaluation included liver function tests, APRI, and FIB-4 scores. Participants underwent ⁶⁸Ga-FAPI PET/CT, and SUVmax, SUVmean, FAPI-avid liver volume (FLV), and total liver FAPI activity (TLFA) were quantified. Correlation and regression analyses were done between PET parameters, biochemical markers, and Child–Pugh classes. Diagnostic performance was assessed using receiver operating characteristic analysis and effect size estimation. Discordance analysis was done for PET-derived fibroblast activity, Child–Pugh classes, and biochemical fibrosis indices.

**Result:**

CLD patients demonstrated significantly raised PET-derived parameters and deranged biochemical markers compared to controls (*p* < 0.05). Biochemical markers worsened progressively across Child–Pugh classes (*p* < 0.05), whereas SUVmax showed a non-significant increasing trend. Compared with controls, Child–Pugh A patients had significantly higher SUVmax, FIB-4, and APRI indices with high discrimination (AUC = 1.00, 1.00, and 0.965, respectively). No significant association was noted between PET parameters, Child–Pugh class, or biochemical fibrosis indices. Only 17% of SUVmax variance was explained by biochemical parameters. Discordance analysis showed High SUVmax in 37.5% and 58.3% of patients with intermediate FIB-4 and mild–moderate APRI categories, respectively. All patients with advanced fibrosis had persistently raised FAPI uptake.

**Conclusion:**

⁶⁸Ga-FAPI PET/CT shows significant fibroblast activation in early CLD with intrahepatic heterogeneity. It shows concordance with the severity of biochemical fibrosis indices at extremes. PET-derived fibroblast activity shows discordance with functional disease severity and intermediate-grade biochemical fibrosis. These findings suggest a complementary role for FAPI PET in characterizing FAP-associated stromal activity beyond conventional biochemical fibrosis indices in CLD.

**Supplementary Information:**

The online version contains supplementary material available at https://doi.org/10.1186/s41824-026-00336-1.

## Introduction

Chronic liver disease (CLD) remains an important global cause of morbidity and mortality and includes non‑alcoholic fatty liver disease, viral etiologies, alcohol‑related liver disease, and others (Zhao et al. 2025, Devarbhavi et al. 2023). CLD refers to persistent (> 6 months) hepatic injury that triggers hepatic stellate cells and portal fibroblasts that differentiate into myofibroblasts. These cells synthesize excessive collagen, leading to progressive fibrosis (Smet et al. 2021, Brenner 2009). Advanced and architectural distortion leads to cirrhosis with variable hepatic dysfunction (Germani et al. 2010). Accurate assessment of fibrosis is essential for CLD staging, prognostication, and management.

Biopsy, biochemical markers, and imaging‑based elastography assess liver fibrosis (Chadha and Sterling 2024). Biopsy remains the reference standard, but is invasive and provides a limited representation of the heterogeneous liver fibrosis (Schiano et al. 2005). Noninvasive blood-based biochemical markers FIB-4 and the AST‑to‑Platelet Ratio Index (APRI) are widely used. They do not directly measure fibrosis and are indirect surrogates of liver fibrosis with variable diagnostic performance (Amorim et al. 2012, Xiao et al. 2015, Graupera et al. 2022). 

Molecular imaging targeting fibroblast activation protein (FAP) has emerged as a promising approach to target activated fibroblasts and FAP-associated stromal activity in chronic tissue injury (Lartey et al. 2025, Hamson et al. 2014). FAP is expressed at low levels in healthy organs. ⁶⁸Ga-FAPI PET/CT is more sensitive than conventional imaging for detecting early fibrosis across various lung, liver, and kidney pathologies (Lartey et al. 2025, Hamson et al. 2014, Aj et al. 2019, A et al. 2024). ⁶⁸Ga-FAPI PET/CT offers the advantage of imaging active fibroblasts and FAP-associated stromal activity in chronic tissue injury. This contrasts with biochemical markers and morphological imaging, which assess established structural fibrosis.

Few small preclinical and human studies have shown that FAPI has low uptake in normal liver parenchyma and increased uptake in CLD. Previous small cohort studies have demonstrated increased hepatic FAPI uptake in CLD and have suggested associations with fibrosis severity. Although available evidence remains limited (Li et al. 2026, Mori et al. 2025, Pirasteh et al. 2022, Rao et al. 2024, Song et al. 2024). Most studies have focused on the presence of fibrosis or stage-wise fibrosis correlations. Discordance between PET-derived fibroblast activity, biochemical fibrosis indices, and functional status has not been systematically examined. Liver fibrosis is a heterogeneous process, and localized sampling methods may not adequately evaluate the full spectrum of liver fibrogenesis. PET/CT imaging may provide complementary information regarding the spatial distribution and quantification of fibroblast activation.

The present study aims to evaluate ⁶⁸Ga-FAPI-04 PET/CT in patients with CLD across a spectrum of functional severity. We evaluated hepatic FAPI uptake across a spectrum of CLD severity and explored its relationship with biochemical fibrosis indices and hepatic functional reserve.

## Materials and method

### Study population

This prospective observation study was conducted in a tertiary care teaching and research hospital from January’2023 to March’2025. It included 33 adult CLD patients of various etiologies across all grades of Child-Pugh classes A-C, representing the entire disease spectrum. Patients with focal liver lesions or other malignancies were excluded. Histopathologic fibrosis staging and elastography were not available. Therefore, fibrosis severity was not directly assessed. Child-Pugh classification was used as a measure of hepatic functional reserve. APRI and FIB-4 were used as biochemical fibrosis indices.

Additionally, 10 individuals without underlying liver disease were included as controls. These subjects underwent Ga-68 FAPI PET/CT scan for other indications. The liver demonstrated normal morphology with no evidence of CLD. The Institutional Ethics Committee approved the study protocol (2023-137-MD-132), and written informed consent was obtained from all participants.

### Biochemical evaluation

All cases and controls underwent liver function tests, including serum bilirubin, aspartate aminotransferase (AST), alanine aminotransferase (ALT), serum albumin, prothrombin time (PT), International Normalized Ratio (INR), and hepatitis viral markers. Biochemical fibrosis indices, the Aspartate Aminotransferase to Platelet Ratio Index (APRI) and the Fibrosis-4 Index (FIB4), were calculated using standard formulae.

### Ga-68 FAPI PET-CT imaging

No specific preparation was required before imaging. All patients received approximately 1.8 MBq/kg body weight of Ga68-FAPI-04 intravenously. After a 30–45 min uptake interval, imaging was performed on an integrated Biograph™ PET/CT scanner (Siemens Healthineers, Germany). A non-contrast CT scan was performed for attenuation correction and anatomical localization from the lower thorax to the mid-thigh region, followed by PET imaging for 2.5 min per bed position. All controls underwent the same procedure, with the scan field extending from the vertex to the mid-thigh region, as determined by the primary clinical indication.

### Image interpretation

All PET/CT images were visually analyzed by two experienced nuclear medicine physicians, who were blinded to clinical and biochemical data. Semi-quantitative assessment of liver uptake was performed using Syngo.via software (Siemens Healthineers). To ensure reproducibility and reliability and minimize observer variability, region-of-interest (ROI) placement was performed in consensus using a standardized semi-automated protocol. Maximum Standard Uptake Value (SUVmax) was defined as the maximum SUVs measured within the liver parenchymal ROI. An ROI was drawn with a SUVmax cut-off of 1.5 to include the entire liver parenchyma in the axial, coronal, and sagittal planes. A threshold of SUV 1.5 was selected as it exceeded the maximum hepatic SUVmax observed in all control subjects (1.3), thereby minimizing inclusion of physiologic background activity. ROI was monitored to ensure full volumetric coverage of liver parenchyma and to exclude extrahepatic physiological tracer activity from adjacent organs. Major intrahepatic vessels and hilar structures were not specifically excluded because no appreciable FAPI uptake was observed in them. SUVmax and SUVmean were recorded from the segmented liver volume. The segmented volume represented the FAPI-avid liver volume (FLV). Total liver FAPI activity (TLFA) was calculated as the product of FLV and SUVmean. FLV and TLFA were exploratory PET-derived volumetric parameters and should not be interpreted as direct measures of histologic fibrosis burden. Blood-pool activity (SUVmax) was measured using a standardized ROI placed within a large vascular structure. Blood-pool FAPI uptake was low and relatively homogeneous in all participants; additional blood-pool metrics were not collected.

### Statistical analysis

The normality of continuous variables was assessed using the skewness Z value (± 3.29). The continuous variables were expressed as means ± standard deviation, and categorical variables as frequencies (%). Parametric tests were used for normally distributed data, while nonparametric tests were used for non-normally distributed data. For within-group analysis, the paired t-test was used for parametric data and the Wilcoxon signed-rank test for non-parametric data.

To evaluate early disease discrimination between CLD patients and controls, receiver operating characteristic (ROC) curve analysis was performed. Area under the curve (AUC) and corresponding 95% confidence intervals were calculated to assess the diagnostic performance of fibrosis indices and PET-derived parameters. Effect size was quantified using Cohen’s d to compare controls and Child–Pugh A patients. These metrics assessed standardized and non-parametric differences independent of sample size. Residual SUV modeling was performed to address potential mathematical coupling between fibrosis scores and biochemical parameters, thereby evaluating PET-derived fibrogenesis independent of biochemical severity. SUVmax was adjusted for biochemical variables incorporated into clinical scoring systems, including AST, platelet count, bilirubin, albumin, and INR, using linear regression.

To explore potential discrepancies between PET-derived FAPI uptake and biochemical fibrosis indices, a discordance analysis was performed in CLD patients only. APRI was categorized as no or minimal, mid to moderate, or advanced fibrosis based on scores of < 0.5, 0.5–1.5, and > 1.5, respectively. FIB-4 was categorized as low, intermediate, and high risk for scores < 1.5, 1.5-3.0, and > 3, respectively. Because no validated hepatic FAPI uptake thresholds currently exist for CLD, the median SUVmax was used as an exploratory stratification threshold for discordance analysis. This threshold is not intended for clinical classification but to examine relationships between PET activity and biochemical fibrosis categories. SUVmax was dichotomized using the median SUVmax of the CLD patients. Discordance was defined as disagreement between biochemical fibrosis and the PET uptake category. The objective was to identify subgroups in whom PET uptake differed from biochemical fibrosis assessment. The Statistical analysis was performed using SPSS (Version 23.0; Armonk, NY: IBM Corp). Selected analyses were performed in Python (version ≥ 3.9) using standard scientific libraries.

## Results

### Baseline clinical and biochemical characteristics

A total of 33 patients with CLD (22 males, 66.6%) and 10 control subjects were included. CLD was associated with Hepatitis C (*n* = 10, 30.3%), Hepatitis B (*n* = 8, 24.2%), alcohol-related liver disease (*n* = 8, 24.2%), NASH (*n* = 6, 18.2%), and Wilson’s disease (1, 3.0%). CLD patients were significantly older than controls (47.09 ± 11.23 vs. 35.80 ± 8.01, *p* = 0.005). CLD patients demonstrated significantly elevated AST, ALT, and bilirubin; elevated APRI and FIB-4 scores; and lower platelet counts and serum albumin levels (*p* < 0.05). Blood pool activity did not differ significantly between patients and controls (median SUVmax 1.0 vs. 0.9, Mann–Whitney *p* = 0.18), highlighting comparable systemic tracer distribution. SUVmax was significantly higher in CLD patients compared with controls. (Table 1) Despite established CLD, a subset of patients demonstrated normal biochemical parameters. Normal AST and ALT were observed in 54.5% and 21.2% of patients, respectively. Serum bilirubin and albumin levels were within normal limits in 33.3% and 36.4% of patients, respectively. Subgroup analysis according to CLD etiology revealed median hepatic SUVmax values of 6.26 in alcohol-related liver disease, 4.6 in hepatitis C, 4.1 in hepatitis B, and 3.9 in NASH. The small size of individual subgroups precluded robust statistical inference.

**Table 1:** Baseline clinical, biochemical, fibrosis indices, and PET parameters in control subjects and CLD patients

Variable	Control (*n* = 10)	CLD (*n* = 33)	*p*-value
Age (years)	35.80 ± 8.01	47.09 ± 11.23	0.005
AST (IU/L)	19.5 (14.5–31.0)	59.0 (45.0–87.0)	< 0.001
ALT (IU/L)	21.5 (13.75–29.5)	36.0 (26.0–64.0)	0.010
Platelet count (×10³/µL)	288 ± 90.6	92.5 ± 46.9	< 0.001
Bilirubin (mg/dL)	0.70 (0.60–0.78)	1.60 (1.20–3.30)	< 0.001
Albumin (g/dL)	4.41 ± 0.26	3.29 ± 0.80	< 0.001
APRI score	0.20 (0.10–0.38)	2.00 (1.00–3.10)	< 0.001
FIB-4 score	0.60 (0.48–0.81)	5.40 (3.29–7.80)	< 0.001
SUVmax	1.10 (0.90–1.27)	4.60 (3.50–6.40)	< 0.001

Continuous variables presented as mean ± SD or median (IQR), depending on distribution. Comparisons were performed using Student’s t-test or Mann–Whitney U test as appropriate

**Table 2:** Distribution of clinical, biochemical, fibrosis indices, and PET parameters across child–pugh classes

Parameter	Child A (*n* = 10)	Child B (*n* = 12)	Child C (*n* = 11)	*p*-value
**Biochemical Parameters**				
AST (IU/L)	45.0 (24.5–46.8)	84.5 (58.0–117.8)	60.0 (49.0–75.5)	**0.007**
ALT (IU/L)	29.5 (19.5–43.8)	57.5 (35.3–73.3)	30.0 (25.0–57.0)	0.131
Platelet (×10³/µL)	97 (88–122)	77 (59–92)	90 (37–151)	0.329
Bilirubin (mg/dL)	1.15 (0.83–1.35)	1.45 (1.18–1.73)	3.80 (2.90–4.95)	**< 0.001**
Albumin (g/dL)	4.20 (3.80–4.38)	3.10 (2.98–3.42)	2.50 (2.35–2.70)	**< 0.001**
INR	1.34 (1.29–1.48)	1.60 (1.45–1.75)	1.96 (1.79–2.28)	**< 0.001**
***Biochemical Fibrotic Indices***				
APRI	1.10 (0.65–1.43)	2.75 (2.22–3.33)	3.00 (1.10–4.95)	**0.012**
FIB-4	3.40 (2.84–5.02)	6.68 (5.21–8.16)	5.28 (3.46–12.18)	**0.047**
***PET Parameters***				
SUVmax	3.38 (2.67–4.15)	6.10 (4.14–6.65)	4.90 (4.17–7.46)	0.069
SUVmean	1.77 (1.68–2.18)	2.17 (1.85–2.76)	1.90 (1.75–2.38)	0.181
SUVpeak	2.29 (1.80–2.60)	3.30 (2.79–4.03)	3.50 (2.88–3.87)	0.086
FAPI-avid liver volume	195.6 (61.0–545.6)	499.3 (235.4–963.8)	563.8 (392.2–676.7)	0.619
Total liver FAPI activity	290.2 (106.5–984.0)	1284.5 (425.4–2261.8)	1164.3 (677.8–1646.9)	0.412

Values are expressed as median (IQR). Overall comparisons across Child–Pugh classes were performed using the Kruskal–Wallis test

**Table 3:** Diagnostic performance and biological effect size for Early disease detection (Control vs. Child Pugh A patients) Receiver operating characteristic (ROC) analysis was used to assess diagnostic performance. Effect sizes were calculated using Cohen’s d with 95% confidence intervals. Perfect discrimination for SUVmax and FIB-4 should be interpreted cautiously given the limited sample size

Variable	AUC (95% CI)	Optimal Cutoff	Sensitivity	Specificity	Cohen’s d (95% CI)
SUVmax	1.0 (1.0–1.00)	1.9	1.00	1.00	2.60 **(**2.10–5.48)
APRI	0.965 (0.86–1.0)	0.60	0.90	1.00	2.01 (1.42–3.70)
FIB-4	1.0 (1.0–1.0)	1.57	1.00	1.00	2.30 (1.73–3.84)

Receiver operating characteristic (ROC) analysis was used to assess diagnostic performance. Effect sizes were calculated using Cohen's d with 95% confidence intervals. Perfect discrimination for SUVmax and FIB-4 should be interpreted cautiously given the limited sample size

**Table 4:** Discordance between PET-derived fibroblast activity and biochemical fibrosis indices in CLD Patients (*n* = 33)

Fibrosis Marker	Category	SUV 1.3–Median	SUV > Median	*p*-value	Cramér’s V
FIB-4	High risk (*n* = 25)	12 (48.0%)	13 (52.0%)	0.758	0.054
	Intermediate risk (*n* = 8)	5 (62.5%)	3 (37.5%)		
APRI	Advanced fibrosis/cirrhosis (*n* = 19)	10 (52.6%)	9 (47.4%)	0.308	0.267
	Mild–Moderate fibrosis (*n* = 12)	5 (41.7%)	7 (58.3%)		
	No or Minimal fibrosis (*n* = 2)	2 (100%)	0 (0%)		

Values are presented as the number of patients with row-wise percentages. SUVmax was dichotomized using the median SUVmax (4.6) into Intermediate PET activity (1.3–4.6) and High PET activity (> 4.6). Discordance analysis excluded control subjects. Associations between PET fibroblast activity and fibrosis grades were assessed using the Chi-square test, and effect size was quantified using Cramér’s V

### Distribution of PET-derived fibroblast activity and fibrotic indices across functional severity

Clinical and biochemical fibrosis markers demonstrated significant worsening across Child–Pugh classes. Progressively increasing levels of bilirubin and INR, and decreasing levels of albumin and INR, were noted (all *p* < 0.05). Both APRI and FIB-4 showed a progressive increase. PET-derived parameters showed an increasing trend, although this did not reach statistical significance (Table 2).

**Fig. 1:**
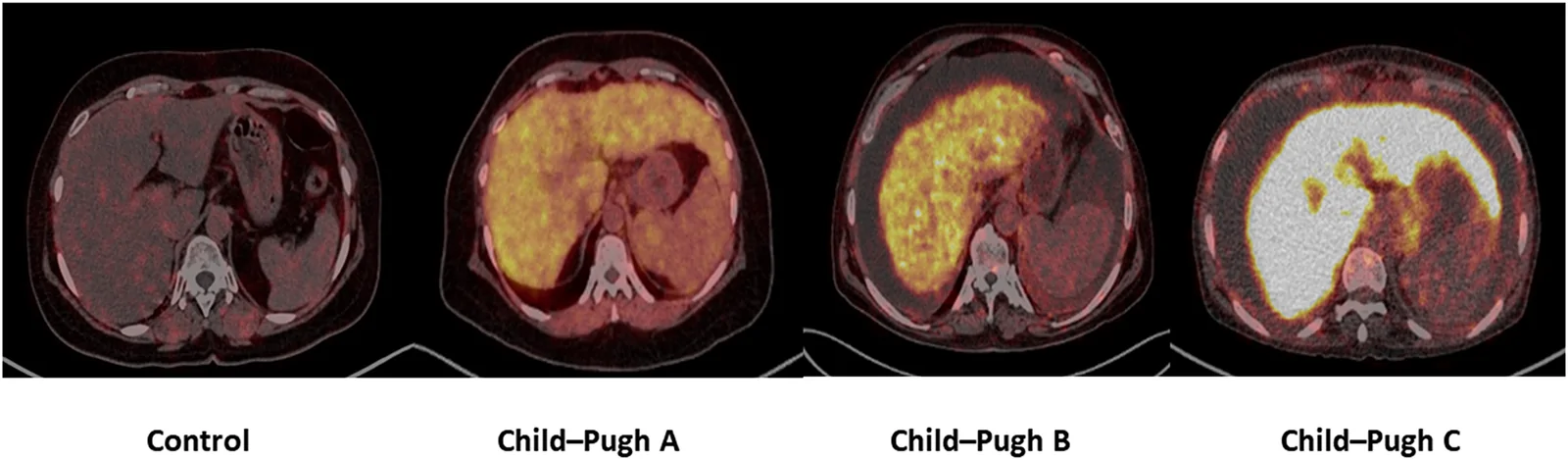
Representative ⁶⁸Ga-FAPI PET/CT images demonstrating hepatic FAPI uptake in a control subject and patients with Child–Pugh A, B, and C CLD. Images show the range of hepatic FAPI uptake observed in the cohort. Uptake was significantly higher in Child–Pugh A patients than in controls; differences among Child–Pugh classes were not statistically significant. The images should not be interpreted as a stepwise increase in hepatic FAPI uptake with Child–Pugh class

**Fig. 2:**
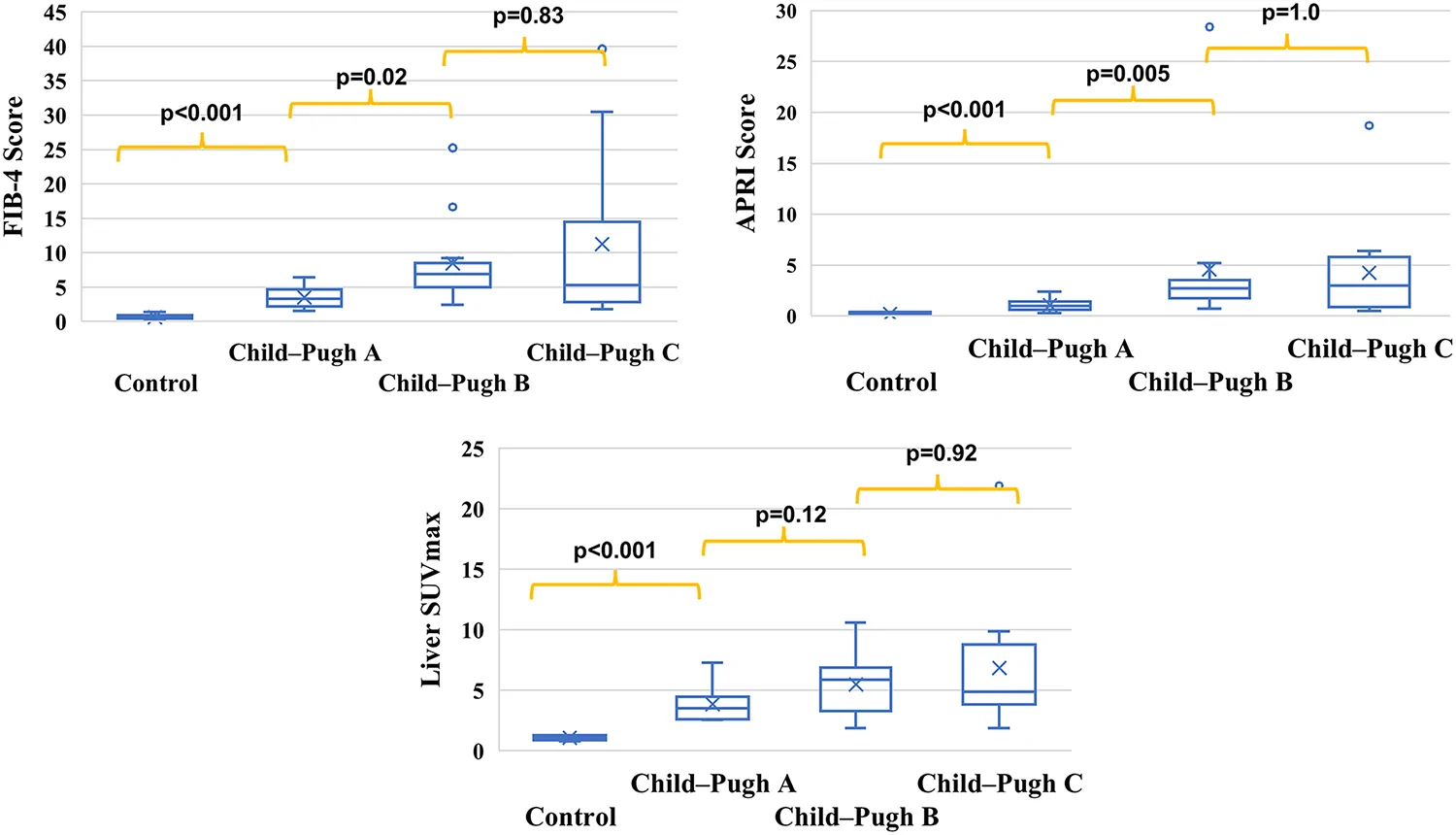
Boxplots demonstrate the distribution of SUVmax, APRI, and FIB-4 across controls and Child–Pugh severity classes. Comparisons were performed using Dunn’s post hoc test with Holm’s correction. SUVmax was significantly elevated in Child–Pugh A patients compared with controls, with a persistent but non-significant elevation in the intermediate and advanced stages. APRI and FIB-4 demonstrate significant progressive increases between early and intermediate disease severity. Note that all three markers show a plateau during intermediate-to-advanced staging

Ordered pairwise comparisons were done between controls and Child–Pugh classes. Child–Pugh A patients showed significantly higher SUVmax than controls (*p* = 0.0005), whereas there were no significant differences among the other Child–Pugh stages. APRI and FIB-4 scores showed significant increases between control and Child–Pugh A (*p* = 0.001 and *p* < 0.001, respectively) and between Child–Pugh A and B (*p* = 0.005 and *p* = 0.022, respectively). These markers reached a plateau with no further significant change between Child–Pugh B and C. Volumetric PET (FLV and TLFA) parameters showed non-significant increases in median values with advancing CLD, highlighting inter-individual variability (Figs. 1 and 2).

**Fig. 3:**
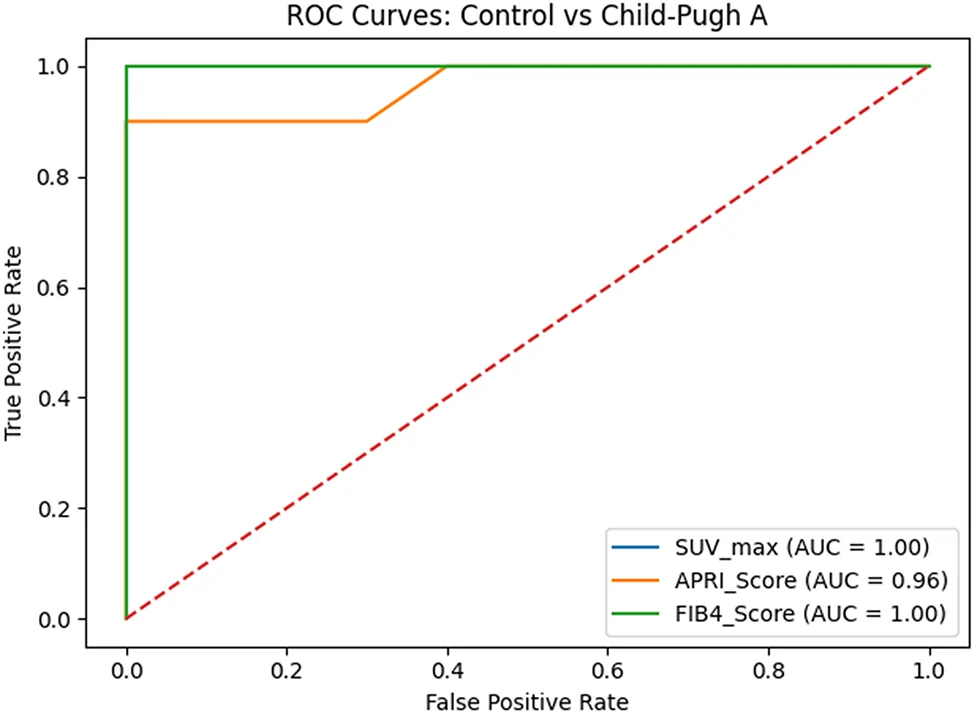
The ROC curve demonstrates the diagnostic performance of PET-derived SUVmax and biochemical fibrosis indices, APRI and FIB-4, in differentiating control subjects from patients with early CLD. All three parameters demonstrated high diagnostic performance

### Association of PET parameters with biochemical factors and Child Pugh scores

PET parameters (SUVmax, FLV, and TLFA) showed no significant correlation with biochemical markers or Child-Pugh scores, including APRI, FIB-4, AST, ALT, platelet count, bilirubin, albumin, PT, and INR (*p* > 0.05; Spearman correlation). In multivariable regression models incorporating SUVmax, SUVratio (SUVmax/SUVbloodpool), FLV, and TLFA, no parameter independently predicted Child–Pugh severity (model *p* = 0.27; adjusted R² = 0.04). APRI demonstrated a significant but moderately positive correlation with Child–Pugh class (Spearman *r* = 0.41, *p* = 0.017). However, FIB-4 showed a non-significant correlation (*r* = 0.32, *p* = 0.07) (Supplement Table 1).

### Detection of early chronic liver disease: control vs. Child–Pugh A

SUVmax and FIB-4 demonstrated perfect discrimination (AUC 1.00) between control and Child–Pugh A patients. APRI also demonstrated high diagnostic performance (AUC 0.965). (Fig. 3) Effect size analysis demonstrated massive standardized differences, with Cohen’s d values of 2.60 for SUVmax, 2.30 for FIB-4, and 2.01 for APRI. (Table 3)Within this subgroup, SUVmax showed no significant correlation with APRI (*r* = − 0.13, *p* = 0.73) or FIB-4 (*r* = 0.22, *p* = 0.54). Adding SUVmax to APRI resulted in a substantial improvement in early disease detection, with a net reclassification improvement of 1.20, driven by correct upward reclassification of 60% of patients and downward reclassification of all controls. However, incremental reclassification beyond FIB-4 was negligible, as FIB-4 alone provided near-perfect discrimination.

As APRI, FIB-4, and Child–Pugh scores are derived from biochemical parameters and may therefore not be statistically independent, they may correlate mathematically. SUVmax was residualized against biochemical parameters incorporated into Child–Pugh classes, APRI, and FIB-4 scores. Only 17% of the SUV variance was explained by AST, platelet, bilirubin, albumin, and INR. Residual SUVmax showed no association with Child–Pugh class (Spearman *p* = 0.26) and had no significant difference between Child–Pugh classes (*p* = 0.081).

### Subset of patients with a normal biochemical profile and FAPI uptake

Four patients (12.1%) had normal AST, ALT, bilirubin, and albumin levels. Despite preserved biochemical profiles, these patients demonstrated elevated hepatic FAPI uptake (SUVmax range 2.6–10.6). Compared with controls, this subgroup showed a very large effect size for SUVmax (Cohen’s d = 2.83). The etiologies were hepatitis B, hepatitis C, alcohol-related liver disease, and NASH, indicating that elevated FAPI uptake was not restricted to a specific one. Three patients were in Child–Pugh class A, and one was in class B. These findings suggest that FAPI uptake may identify FAP-associated stromal activity despite normal biochemical parameters.

### Discordance between child pugh scores, FAPI PET, and biochemical fibrosis indices

- Functional Discordance (Supplement Table 2).

Cross-tabulation analysis demonstrated a significant association between Child–Pugh class and APRI grade (*p* = 0.046). However, the FIB-4 grade did not show a significant association with Child–Pugh class (*p* = 0.216). Median-split SUVmax demonstrated a trend toward association with functional severity (*p* = 0.082). This suggests that the functional decompensation is not solely dependent on FAP-associated stromal activity.

- Fibrosis Discordance (Table 4).

Discordance analysis was performed in CLD patients with intermediate SUVmax (1.3 to the median SUVmax) and high SUVmax (above the median SUVmax). Concordance was observed at the extremes of biochemical fibrosis assessment. Patients with minimal fibrosis generally showed intermediate PET uptake, whereas those with advanced fibrosis consistently showed elevated PET uptake. Only two patients with minimal fibrosis on FIB-4 demonstrated intermediate SUVmax values. However, substantial heterogeneity was observed within the intermediate fibrosis group. 37.5% patients with intermediate FIB-4 grade and more than half (58.3%) of patients with mild–moderate APRI exhibited high SUVmax on FAPI PET/CT. These suggest that FAPI uptake may further stratify patients within intermediate biochemical fibrosis categories. Among advanced fibrosis patients, no physiologic SUVmax values (< 1.3) were observed, indicating persistent fibroblast activation even in advanced disease.

## Discussion

FAP is expressed by activated fibroblasts and hepatic stellate cells, which are involved in extracellular matrix remodeling during chronic liver injury. However, the biological significance of uptake remains incompletely understood. In the absence of histopathologic validation, the current study cannot directly determine whether FAPI uptake reflects fibrosis burden, active matrix deposition, inflammation-associated uptake, or tissue remodeling. FAPI uptake should be interpreted as a marker of FAP-associated stromal activity rather than a direct measure of liver fibrosis.

### Principal findings and biological interpretation

This prospective study evaluated ⁶⁸Ga-FAPI PET/CT in 33 CLD patients with variable functional grades and 10 control subjects. CLD patients demonstrated significantly deranged biochemical parameters, biochemical fibrotic scores, and FAPI-derived parameters compared with controls. Biochemical markers demonstrated progressive worsening across Child–Pugh classes. Among the PET-derived parameters, only SUVmax showed a non-significant rising trend with functional disease severity. Biochemical fibrotic markers, as well as SUVmax, showed a plateau between Child–Pugh B and C classes. Marked heterogeneity was noted between PET-derived fibroblast activity and biochemical fibrotic markers in intermediate fibrosis categories. Exploratory etiology subgroup analysis demonstrated only modest variation in FAPI uptake.

FAPI PET interrogates selective FAP expression by activated fibroblasts during fibrogenesis. While biochemical fibrosis indices indirectly estimate fibrosis. This biological specificity may explain the limited correlation observed between FAPI-PET and biochemical fibrosis scores.

### Detection of early chronic liver disease

Both FAPI PET and FIB-4 demonstrated excellent discrimination between Child–Pugh A patients and control subjects, with large effect sizes. However, adding SUVmax to APRI improved the classification of early disease, indicating a complementary role. A subset of patients with normal biochemical parameters exhibited elevated PET activity, suggesting persistent FAP-associated stromal activity despite normal biochemical parameters. Residual SUVmax analysis demonstrated that FAPI PET provided biological information beyond that explained by biochemical markers alone. A particularly interesting finding was elevated hepatic FAPI uptake in four Child–Pugh A patients despite normal liver biochemistry. The observation was not disease-specific. Although the significance of this finding requires histopathologic validation, this observation suggests that molecular imaging may reveal FAP-associated stromal activity that is not fully captured by biochemical assessment.

Activated hepatic stellate cells in fibrosis express FAP, which is absent in normal hepatocytes, resulting in very low FAPI uptake in controls. The existing preclinical and clinical studies suggest that FAPI PET/CT detects early fibrosis, with a gradual increase in uptake across CLD stages. In a study including 14 participants who underwent liver biopsy using the METAVIR scoring system, the FAPI liver-to-blood ratio increased gradually from fibrosis grades F0/1 to F4 (0.83 ± 0.07 vs. 2.12 ± 0.78, *P* = 0.010). Ga-FAPI-04 PET/CT differentiated fibrosis grades in 85.7% of participants (Li et al. 2026). Preclinical animal studies with CCl4-induced CLD have shown a strong correlation between fibrosis stage and SUVmax (*r* = 0.65–0.89). High FAPI uptake is noted in human subjects with advanced fibrosis compared with controls (Song et al. 2024). Small cohort studies have shown a promising role for FAPI-04 PET/CT in evaluating early-stage liver fibrosis in liver transplant recipients (Rao et al. 2024). However, in animal models, FAPI-46 PET/MRI failed to demonstrate a difference between baseline and F0/F1 fibrosis (Pirasteh et al. 2022). 

### Liver function vs. liver fibrosis

PET parameters and FIB-4 did not correlate significantly with Child-Pugh score severity. At the same time, APRI had a moderately positive correlation with Child–Pugh class. Similar to our study, Mori et al. observed a significant but weak correlation between SUVmax and APRI score (*r* = 0.163, *P* = 0.021), with no correlation between FAPI uptake and the FIB-4. A significant increase in SUV parameters was observed across rising APRI scores (< 0.5, 0.5–1.0, and > 1.0) (Mori et al. 2025). The liver has a large functional volume, and hepatic function may remain preserved until advanced fibrosis. These findings indicate that fibroblast activity is not perfectly interrelated to functional decompensation.

### Liver fibrosis: concordance at extremes and heterogeneity in intermediate fibrosis

Among patients with minimal or advanced fibrosis, concordance between biochemical fibrotic markers and SUVmax was observed. However, in the intermediate fibrosis group, significant discordance was observed. This suggests that fibroblast activity may further stratify this subgroup. No patients with advanced fibrosis demonstrated physiologic SUVmax, suggesting persistent fibrogenesis.

Several studies have shown the limitations of biochemical markers in assessing liver fibrosis. variable sensitivity of APRI for detecting mild, advanced fibrosis, and cirrhosis (70%, 50%, and 36.9%, respectively), as well as FIB-4 for detecting mild and advanced fibrosis (65.4% and 16.2%, respectively) in hepatitis B (Xiao et al. 2015). In hepatitis C, APRI and FIB-4 showed similar moderate performance (Amorim et al. 2012). In a large multicenter trial, including 5129 patients who underwent FIB-4, NFS, and Fibroscan, 28% of patients with elevated FIB-4 were false positives (Graupera et al. 2022). In a recent prospective pilot study including 14 CLD patients, METAVIR biopsy staging was compared with FAPI-04 PET/CT, Fibroscan, and FIB-4. FAPI used TBR liver-to-blood thresholds: <1 (F0/1), 1–2 (F2/3), > 2 (F4), achieving 85.7% accuracy, compared with 71.4% for both Fibroscan and FIB-4 (Li et al. 2026). In the current study, hepatic FAPI uptake demonstrated limited correlation with APRI, FIB-4, and Child–Pugh class, and only 17% of SUVmax variance was explained by biochemical parameters. These findings suggest that FAPI uptake reflects biological processes that are not fully captured by conventional biochemical assessment. Elevated FAPI uptake in a subset of patients with normal AST, ALT, bilirubin, and albumin levels further supports this hypothesis.

Liver biopsy remains the gold standard for assessing liver fibrosis. However, it represents only a fraction of the liver parenchyma, potentially leading to an underestimation of liver fibrosis (Schiano et al. 2005). PET provides a whole-liver assessment of fibroblast activity and may complement or guide biopsy sampling. The weak, insignificant correlation among FIB-4, APRI, and FAPI-PET parameters supports the idea that FAPI-PET imaging targets stromal activity. PET interrogates a biological process that appears partially independent of biochemical fibrosis indices and hepatic functional reserve.

### Clinical implications, limitations, and future directions

The findings of this study suggest that ⁶⁸Ga-FAPI PET/CT may play a complementary role to conventional biochemical fibrosis markers and biopsy in the early detection of fibroblast activation in CLD patients with near-normal biochemical parameters. Fibroblast activity may further provide biological stratification within intermediate fibrosis categories. It could have clinical implications for risk stratification and therapeutic decision-making.

A single-center design, a small sample size, and subgroup analyses may limit the generalizability of the study. The etiologies of CLD were varied. Histopathologic confirmation and liver stiffness measurements were unavailable. Consequently, the liver fibrosis stages were not directly established. APRI and FIB-4 were used as surrogate biochemical fibrosis indices. Therefore, the present study cannot determine whether hepatic FAPI uptake reflects fibrosis burden, tissue remodeling, or inflammation-associated stromal activation. Interobserver reproducibility was not evaluated and should be addressed in future studies. Larger etiology-specific studies are required to determine whether viral, metabolic, and alcohol-related liver diseases demonstrate distinct patterns of FAP-associated stromal activity. The study utilized Blood pool SUVmax, which is less commonly used for PET normalization. Future studies should incorporate standardized blood-pool SUVmean measurements. Prospective, longitudinal studies with histopathological correlation are required to determine whether FAPI uptake and heterogeneity could access fibrotic stage, disease progression, or therapeutic response. Integration of FAPI PET-derived parameters with biochemical markers may improve individualized risk stratification. Further research could explore monitoring fibroblast activation and its response to antiviral or antifibrotic therapies, as dynamic changes may serve as biomarkers of treatment response. Standardization of acquisition protocols and quantification is essential for broader applications.

## Conclusion

⁶⁸Ga-FAPI PET/CT demonstrates no significant hepatic uptake in healthy controls. It shows increased uptake in CLD with weak correlation with biochemical markers and functional status. Significant discordance is noted in the intermediate fibrosis categories. These findings support a complementary role for FAPI PET in disease stratification. Prospective studies with histopathological correlation and longitudinal follow-up are required to characterize fibrosis heterogeneity, identify early fibrogenesis in which conventional fibrotic markers are negative, and support prognostication.

## Supplementary Information

Below is the link to the electronic supplementary material.

Supplementary Material 1

## Data Availability

The datasets generated and/or analyzed during the current study are not publicly available due to institutional data protection policies and patient confidentiality requirements, but are available from the corresponding author on reasonable request.
