# Supplementary material for: ⁶⁸Ga-FAPI PET/CT reveals fibroblast activation beyond biochemical fibrosis scores in chronic liver disease
Source: EJNMMI Rep. 2026 Nov 13;10(1):47. doi: 10.1186/s41824-026-00336-1 (PMC13759964; doi:10.1186/s41824-026-00336-1)
Supplement: Supplementary file 1 [file 41824_2026_336_MOESM1_ESM.docx]

| **Supplementary Table 1. Correlation Between PET Parameters and Biochemical Fibrosis Indices** | | | |
| --- | --- | --- | --- |
| **PET Pasrameter** | **Score** | **Spearman r** | **p-value** |
| Gross FibroticVolume | APRI | 0.110 | 0.543 |
| Gross Fibrotic Volume | FIB-4 | 0.133 | 0.461 |
| Total Liver Fibrosis | APRI | 0.045 | 0.802 |
| Total Liver Fibrosis | FIB-4 | 0.107 | 0.555 |
| SUVmax | APRI | 0.102 | 0.573 |
| SUVmax | FIB-4 | 0.193 | 0.282 |
| Correlations were assessed using Spearman's rank correlation analysis. | | | |

| **Supplementary Table 2: Distribution of Fibrosis Grades and PET-Derived Fibroblast Activity Across Functional Status (Child–Pugh Classes)** | | | | | |
| --- | --- | --- | --- | --- | --- |
| **Parameter** | **Category** | **Child Pugh Class** | | |  |
|  |  | **Child A (n=10)** | **Child B (n=12)** | **Child C (n=11)** | **p-value** |
| **APRI Grade** | Advanced fibrosis/cirrhosis | 2 (10.5%) | 10 (52.6%) | 7 (36.8%) | **0.0459** |
|  | Mild–Moderate Fibrosis | 7 (58.3%) | 2 (16.7%) | 3 (25.0%) |  |
|  | No or Minimal | 1 (50.0%) | 0 (0.0%) | 1 (50.0%) |  |
| **FIB-4 Grade** | High risk | 6 (24.0%) | 11 (44.0%) | 8 (32.0%) | 0.2165 |
|  | Intermediate risk | 4 (50.0%) | 1 (12.5%) | 3 (37.5%) |  |
| **SUVmax (Median Value)** | High | 2 (12.5%) | 8 (50.0%) | 6 (37.5%) | 0.0821 |
|  | Low | 8 (47.1%) | 4 (23.5%) | 5 (29.4%) |  |
| Values are presented as the number of patients with row-wise percentages. Associations were assessed using the chi-square test. SUVmax was dichotomized using the cohort median. | | | | | |
